# Risk estimation of cardiovascular diseases using the World Health Organization/International Society of Hypertension risk prediction charts in the Azar cohort population: Cross-sectional study

**DOI:** 10.34172/jcvtr.32906

**Published:** 2024-06-25

**Authors:** Elnaz Faramarzi, Mohammad Hossein Somi, Alireza Ostadrahimi, Roghayeh Molani-Gol, Zhila Khamnian, Samad Ghaffari, Bita Amiri

**Affiliations:** ^1^Liver and Gastrointestinal Diseases Research Center, Tabriz University of Medical Sciences, Tabriz, Iran; ^2^Nutrition Research center, Tabriz University of Medical Sciences, Tabriz, Iran; ^3^Student Research Committee, Tabriz University of Medical Sciences, Tabriz, Iran; ^4^Department of Community and Family Medicine, Tabriz University of Medical Sciences, Tabriz, Iran; ^5^Cardiovascular Research Center, Tabriz University of Medical Sciences, Tabriz, Iran

**Keywords:** Cardiovascular disease, WHO, ISH, Risk prediction charts, Azar cohort

## Abstract

**Introduction:**

Cardiovascular disease (CVD) is one of the most important health problems and the leading cause of mortality worldwide. This study aimed to estimate the risk of CVD using the World Health Organization/International Society of Hypertension (WHO/ISH) risk prediction charts.

**Methods:**

The demographic characteristics of all participants of this study aged 40-70 years who did not have a prior coronary event were collected. The 10-year CVD risk was estimated using the laboratory version of the WHO/ISH risk score charts. The risk scores for 11678 participants of the Azar cohort population were calculated. Participants were classified as low risk, moderate risk, or high risk.

**Results:**

According to the WHO/ISH charts, only 0.1 % of the population was classified as high-risk (≥40%), and 96.8% had a 10-year CVD risk of<10%. Also, participants with overweight (*P*=0.002), obesity, and abdominal obesity had higher CVD risk(*P*<0.001).

**Conclusion:**

There was a low burden of 10-year CVD risk among the Azar cohort population without prior coronary events. It appears the percentage of people in the high-risk group is underestimated in the WHO/ISH risk prediction charts, leading to delays in receiving appropriate management in the population concerned. Therefore, using other charts alongside the WHO/ISH risk prediction charts is advisable.

## Introduction

 Cardiovascular disease (CVD) is one of the most important health problems and the leading cause of mortality worldwide.^1^ It is the main reason for disability and the most predictable non-communicable diseases that significantly reduce the quality of life.^1^ Based on the 2019 global Burden of Disease (GBD) report, CVD remains the primarycause of death in Iran, followed by stroke and injuries from road accidents.^2^ Classical risk factors of CVD, such as hypertension, high cholesterol, diabetes mellitus, obesity, and smoking, have been used to create risk scores that can predict the likelihood of events such as myocardial infarction and stroke.^3^ These scores have been used in recent guidelines to aid physicians in taking appropriate primary and secondary preventive measures; for example, individualized Statin therapy based on ten-year CVD risk is one of the risk-score guided treatments.^4^

 Some of the most well-known risk scores are Framingham Risk Score-CVD (FRS-CVD), American College of Cardiology/American Heart Association (ACC/AHA), Atherosclerotic CVD (ASCVD), and World Health Organization (WHO) risk charts. Studies that have compared the performances of these risk scores in different populations indicate that the estimation power drops sharply in dissimilar populations.^5,6^ Therefore, patients should be treatedonly based on their population-validated risk scores.^7^

 The WHO/International Society of Hypertension (ISH) risk prediction charts are a series of color-coded charts recommended by WHO guidelines for CVD prevention. Different charts are available for the various WHO epidemiological sub-regions around the world.^8,9^ There is limited information about the accuracy or validation procedure of the WHO/ISH risk prediction charts in literature; the charts were not developed using prospective or out-of-sample test data, and the methods employed differ from other risk estimation functions.^10^

 Various studies have calculated CVD risk using different risk scores in the Iranian population,^11-13^ and a few have used WHO risk prediction charts.^14^ Since these risk factors are easily modifiable, their distributions are investigated globally through large cohorts like the Azar cohort’s current study. As no previous studies have evaluated the 10-year risk in the Azar cohort population, we aimed to assess the 10-year risk of CVD in 40 to 70-year-old participants through a cross-sectional survey for the first time.This information will assist significantly in the planning and management of non-communicable diseases. Therefore, our study aims to evaluate the risk of CVD in a population-based study using the WHO risk prediction charts and to identify factors associated with CVD risk in the Azar cohort population.

## Materials and Methods

 Thiscross-sectional study used the data fromthe Azar cohort study. The Azar cohort study^15^ is one of several ongoing studies of the large Prospective Epidemiological Research Studies in Iran (PERSIAN cohort).^16^ The enrollment phase of this cross-sectional study was conducted from 2014 to 2017. The inclusion criteria of the present study comprised subjects aged 40-70 who consented to participate. The exclusion criteria were a history of CVD, Myocardial infarction, stroke, psychological or mental health, developmental issues, and hearing or vision loss that made cooperation problematic. Moreover, subjects with missing valuesin CVD risk estimation components were excluded. According to our inclusion criteria, 11678 participants were involved in the present study. Informed consent was obtained from all participants in the study. The present study has been approved by the Ethical Committee of the University of Medical Sciences (ethical code: IR.tbzmed.rec.1399.877).

###  Demographic characteristics 

 An available questionnaire was used to obtain demographic information from participants, such as age, sex, marital status, and educational background. Personal behaviors like smoking were assessed using a questionnaire. Socioeconomic status was evaluated using the Wealth Score Index (WSI), calculated using Multiple Correspondence Analysis (MCA). Ownership of various durable assets (e.g., dishwasher, car, and television), household condition (e.g., the number of rooms, type of ownership), and education level were used to calculate WSI for each participant. Study participants were categorized into five SES quintiles, from the lowest (1st quintile) to the highest (5th quintile). In this study, the participants’ daily activities were determined using a questionnaire recorded by the participants. For this purpose, a criterion called MET has been employed. One metabolic equivalent (MET) equals the amount of energy each person consumes relative to their weight. For instance, one MET equals the amount of oxygen each person uses while resting per kilogram of their body weight per minute, which is 3.5 ml of oxygen, and 4 MET equals 14 milliliters of oxygen used per kilogram of their body weight per minute. Through this criterion, we obtained the activity level based on each person’s respective MET.

###  Anthropometric and blood pressure (BP) measurements

 The weight and height of all subjects were measured, and the body mass index (BMI) was determined using the standard formula: weight (kg)/height (m2). BMI was classified according to the WHO categories: underweight (BMI < 18.5 kg/m2), normal (BMI = 18.5 - 24.9kg/m2), overweight (BMI = 25-29.9 kg/m2), and obesity (BMI ≥ 30 kg/m2). Subjects’ waist circumference (WC) and hip circumference are measured according to National Institute of Health (NIH) guidelines. Waist-to-hip ratio (WHR) and waist-to-height ratio (WHtR) were calculated by dividing waist-to-hip and height, respectively. In our participants, WC ≥ 102 cm in males and ≥ 88 cm in females, WHR ≥ 0.9 in males and ≥ 0.85 in females, and WHtR ≥ 0.5 were considered as abdominal obesity indices. BP was measured twice, at approximately 10-minute intervals, after the participant had rested in a seated position for 15 minutes using a Richtersphygmomanometer.

###  Risk estimation of CVD using the WHO criteria

 The study employed WHO/ISH risk prediction charts, which estimatea 10-year risk of a fatal or nonfatal significant CVD event (myocardial infarction or stroke). It includes age (years), gender, SBP (mmHg), total cholesterol mg/dl (TC), smoking status, and the presence or absence of diabetes mellitus for the various WHO epidemiological sub-regions. We applied the Eastern Mediterranean B subgroup (EMR B) chart, which is region, country, and income-specific (WHO, 2007).^9^ For total CVD risk calculation, a smoker is currently smoking or has quit smoking for less than a year, and diabetes is defined as fasting blood glucose ≥ 126 mg/dl or a participant on diabetes medication. A total of 5 mlof blood (one clot tube and three with EDTA) was sampled from every participant. All samples were taken after 12-14 hours of fasting between 7 and 9 A.M. Lipid profiles were measured using a Pars Azmoon kit.

###  Statistical Analysis

 A descriptive analysis was conducted for basic characteristics in the studied population. The normality of data was evaluated by Kurtosis and Skewness indices. All measurements were expressed as mean ± standard deviation (SD) or Median (interquartile range) and percentages for categorical variables. Comparisons between categorical variables were performed using the chi-square (χ2) test or Kruskal Wallis, One-way ANOVA, and independent t-test for continuous variables.The score of 10-year CVD risk was classified into two groups: < 10% and ≥ 10 %. Binary logistic regression was used to evaluate risk factors of 10-year CVD risk; in this analysis, a group with a < 10% risk score was considered a reference group. Multivariable logistic regression (adjusted for age, and gender) with 10% risk score as dependent variable and each risk factor as independent variable was used to estimate the adjusted odds ratio and 95% confidence interval. Statistical significance has been defined as a two-tailed probability of < 0.05 in this study. SPSS v. 20 (SPSS Inc., Chicago, IL, USA) was used for all analyses.

## Results

 According to the aforementioned inclusion criteria, 11678of 15006 participants were included, and 69% were from urban. The mean age (years)of participants was 51.74 (SD = 7.94), and 55.07% (6432) of them were female. More than 40% of our participants were overweight, and 38.7% of them were obese. Other demographic characteristics of the participants and risk factors of CVD by gender with comparison are presented in Table 1. Using theWHO-Eastern Mediterranean Region (WHO-EMR) B model, 96.8% of the population had a 10-year CVD risk estimate of < 10% and was considered low risk. Only 12 participants (0.1%) had a risk estimate of ≥ 40% (Table 1). Of 96.8% of participants that had < 10% CVDrisk, 40.8% were overweight, and 38.3% of them were obese. Also, among them, 13.5% were smokers, and 11.4 and 19.4% had diabetes and hypertension, respectively (Table 2).

**Table 1:** Demographic characteristics of study population

**Variables**	**Total (n=11678)**	**Male (n=5246)**	**Female (n=6432)**	*P** value**
	**N (%)**	**N (%)**	**N (%)**	
**Education level **				
Illiterate	2238 (19.2)	514 (9.8)	1724 (26.8)	< 0.001
Primary school	4948 (42.4)	2085 (39.7)	2863 (44.5)	
Diploma	3576 (30.6)	2049 (39.1)	1527 (23.7)	
University	916 (7.8)	598 (11.4)	318 (4.9)	
**Residential regions**				0.02
Urban	8058 (69)	3675 (70.1)	4383 (68.2)	
Rural	3620 (31)	1571 (29.9)	2049 (31.9)	
**Physical activity level (**^$^**METs)**				
Low	3808 (32.6)	1558 (29.7)	2250 (35.0)	< 0.001
Moderate	3916 (33.5)	1048 (20)	2868 (44.6)	
High	3954 (33.9)	2640 (50.3)	1314 (20.4)	
**Quintiles of wealth index**				
1 (poorest)	2429 (20.8)	758 (14.4)	1671 (26)	< 0.001
2	2364 (20.3)	1015 (19.3)	1349 (21)	
3	2249 (19.3)	1072 (20.4)	1177 (18.3)	
4	2452 (21)	1159 (22.1)	1293 (20.1)	
5 (richest)	2184 (18.7)	1242 (23.7)	942 (14.7)	
**Categorical Risk **				
< 10	11299 (96.8)	5086 (97.0)	6213 (96.6)	0.21
10-19	270 (2.3)	116 (2.2)	154 (2.4)	
20-29	70 (0.6)	23 (0.4)	47 (0.7)	
30-39	27 (0.2)	14(0.3)	13 (0.2)	
≥ 40	12 (0.1)	7 (0.1)	5 (0.1)	
Smoking	1581 (13.5)	1548 (29.5)	33 (0.5)	< 0.001
**Marital status **				< 0.001
Not married	861 (7.4)	41 (0.8)	820 (12.7)	
Married	10817 (92.6)	5205 (99.2)	5612 (87.3)	
**Past history**				
Diabetes	1452 (12.4)	561 (10.7)	891 (13.8)	< 0.001
Hypertension	2428 (20.8)	715 (13.6)	1713 (26.6)	< 0.001
^£^**BMI (kg/m**^2^**)**				
Underweight (BMI < 18.5)	75 (0.7)	57 (1.1)	18 (0.3)	< 0.001
Normal weight (BMI = 18.5-24.9)	2330 (19.9)	1434 (27.3)	896 (13.9)	
Overweight (BMI = 25-29.9)	4755 (40.7)	2396 (45.7)	2359 (36.7)	
Obese (BMI ≥ 30)	4518 (38.7)	1359 (25.9)	3159 (49.1)	
**Waist circumference (cm) **				< 0.001
< 102male; < 88 female	5586 (47.8)	3805(72.5)	1781 (27.6)	
≥ 102 male; ≥ 88 female	6090 (52.2)	1440 (12.3)	4650 (72.4)	
**Waist-to-hip ratio**				0.08
< 0.9 male; < 0.85 female	3625 (31.1)	1586(30.2)	2039 (31.7)	
≥ 0.9 male; ≥ 0.85 female	8051 (68.9)	3659 (69.8)	4392 (68.3)	
**Waist to height ratio **				< 0.001
< 0.5	1367(11.7)	893(17)	474(7.4)	
≥ 0.5	10309 (88.3)	4352 (83)	5957 (92.6)	
	**Mean±SD**	**Mean±SD**	**Mean±SD**	***P***
Age (years)	51.74 ± 7.94	52.04 ± 7.92	51.49 ± 7.97	< 0.001
Height (cm)	161.84 ± 9.40	169.59 ± 6.56	155.51 ± 6.02	< 0.001
Weight (kg)	75.74 ± 13.59	79.08 ± 13.51	73.02 ± 13.05	< 0.001
BMI (kg/m^2^)	28.95 ± 4.90	27.45 ± 4.27	30.17 ± 5.06	< 0.001
^$$^WC (cm)	94.76 ± 11.17	95.55 ± 11.11	94.11 ± 11.17	< 0.001
Waist to hip ratio	0.90 ± 0.07	0.93 ± 0.06	0.88 ± 0.07	< 0.001
Waist to height ratio	0.58 ± 0.07	0.56 ± 0.06	0.60 ± 0.07	< 0.001
^¥^SBP (mmHg)	115.09 ± 17.26	114.439 ± 17.19	115.63 ± 17.31	< 0.001
^¥¥^DBP (mmHg)	74.10 ± 9.74	74.124 ± 9.79	74.08 ± 9.71	0.82
^#^FBS (mg/dl)	100.43 ± 33.18	99.31 ± 31.15	101.34 ± 34.73	0.009***
Median (interquartile rang)	92 (19)	91 (18)	92 (20)	
Cholesterol (mg/dl)	196.37 ± 39.99	189.62 ± 38.20	201.88 ± 40.58	< 0.001
Triglyceride (mg/dl)	151.35 ± 85.12	156.27 ± 92.80	147.34 ± 78.02	< 0.001***
Median (interquartile rang)	126 (76)	129 (83)	124 (71)	
^€^LDL (mg/dl)	119.71 ± 34.24	116.15 ± 33.20	122.61 ± 34.80	< 0.001
^€€^HDL (mg/dl)	46.46 ± 11.04	42.34 ± 9.47	49.81 ± 11.10	< 0.001

^$^MET: Metabolic equivalent of task; ^£^BMI: Body mass index;^$$^WC: Waist circumference; ^¥^SBP: Systolic blood pressure; ^¥¥^DBP: Diastolic blood pressure;^#^FBS: Fasting blood sugar; ^€^LDL: Low density lipoprotein; ^€€^HDL: High density lipoprotein;* P: Chi square test; ^**^P: Independent samples T-test; ***P: Mann-Whitney U

**Table 2:** Study population characteristics based on 10-year cardiovascular risk

**Variables**	**<10 %**	**10-19 %**	**20-29 %**	**30-39 %**	**>40 %**	**P** value**
	**N (%)**	**N (%)**	**N (%)**	**N (%)**	**N (%)**	
**Residential regions**						
Urban	7858 (69.5)	144 (53.3)	38 (54.3)	13 (48.1)	5 (41.7)	< 0.001^*^
Rural	3441 (30.5)	126 (46.7)	32 (45.7)	14 (51.9)	7 (58.3)	
**Education level**						< 0.001
Illiterate	2066 (18.3)	121 (44.8)	37 (52.9)	9 (33.3)	5(41.7)	
Primary school	4824 (42.7)	86 (31.9)	23 (32.9)	9 (33.3)	6(50)	
Diploma	3503 (31)	54 (20)	10 (14.3)	8 (29.6)	1(8.3)	
University	906 (8)	9 (3.3)	0	1 (3.7)	0	
^¥^**Physical activity level (METs)**						< 0.001
Low	3640(32.2)	122 (45.2)	31(44.3)	12 (44.4)	3 (25)	
Moderate	3804(33.7)	76 (28.1)	24(34.3)	8 (29.6)	4 (33.3)	
High	3855(34.1)	72 (26.7)	15 (21.4)	7 (25.9)	5 (41.7)	
**Quintiles of wealth index**						< 0.001
1 (poorest)	2308 (20.4)	81 (30)	27 (38.6)	9 (33.3)	4 (33.3)	
2	2244 (19.9)	85 (31.5)	21(30)	7 (25.9)	7 (58.3)	
3	2185(19.3)	48 (17.8)	12(17.1)	4 (14.8)	0	
4	2420 (21.4)	21 (7.8)	7 (10)	4 (14.8)	0	
5 (richest)	2142 (19)	35 (13)	3 (4.3)	3 (11.1)	1 (8.3)	
Smoking	1524 (13.5)	43 (15.9)	7 (10)	5 (18.5)	2 (16.7)	0.59^*^
**Marital status **						
Not married	809 (7.2)	32 (11.9)	15 (21.4)	5 (18.5)	0	< 0.001^*^
Married	10490 (92.8)	238 (88.1)	55 (78.6)	22 (81.5)	12 (100)	
Past history						
Diabetes	1290 (11.4)	108 (42.2)	36 (54.5)	12 (52.2)	6 (60)	< 0.001^*^
Hypertension	2189 (19.4)	152 (56.3)	54 (77.1)	23 (85.2)	10 (83.3)	< 0.001^*^
^€^**BMI (kg/m**^2^**) **						< 0.001
Underweight < 18.5	73 (0.6)	2 (0.7)	0	0	0	
Normal weight 18.5-24.9	2280 (20.2)	45 (16.7)	4 (5.7)	0	1 (8.3)	
Overweight 25-29.9	4613 (40.8)	111 (41.1)	15 (21.4)	11 (40.7)	5 (41.7)	
Obese ≥ 30	4333 (38.3)	112 (41.5)	51 (72.9)	16 (59.3)	6(50)	
**Waist circumference (cm) **						< 0.001*
< 102male; < 88 female	5468(48.4)	98(36.3)	10(14.3)	6(22.2)	4(33.3)	
≥ 102 male; ≥ 88 female	5829(51.6)	172(63.7)	60 (85.7)	21(77.8)	8(66.7)	
**Waist-to-hip ratio**						< 0.001*
< 0.9 male; < 0.85 female	3589 (31.8)	27(10.0)	6(8.6)	2(7.4)	1(8.3)	
≥ 0.9 male; ≥ 0.85 female	7708(68.2)	243(90)	64(91.4)	25(92.6)	11(91.7)	
**Waist-to-height ratio **		0.07				< 0.001*
< 0.5	1355(12)	12(4.4)	0	0	0	
≥ 0.5	9942(88)	258(95.6)	70(100)	27(100)	12(100)	
	**Mean±SD**	**Mean±SD**	**Mean±SD**	**Mean±SD**	**Mean±SD**	***P*** ***
Age (years)	51.37 ± 7.74	62.57 ± 5.766	62.57 ± 5.43	62.59 ± 6.59	62.08 ± 3.80	< 0.001
Height (cm)	161.94 ± 9.38	159.05 ± 9.12	157.42 ± 9.62	159.43 ± 9.91	160.71 ± 7.87	< 0.001
Weight (kg)	75.72 ± 13.54	74.59 ± 14.73	80.99 ± 16.83	80.50 ± 14.68	75.33 ± 9.86	0.005
BMI (kg/m^2^)	28.90 ± 4.90	29.55 ± 4.94	32.51 ± 5.05	31.57 ± 4.33	29.06 ± 2.13	< 0.001
Waist circumference (cm)	94.58 ± 11.13	98.79 ± 11.05	103.69 ± 11.06	104.30 ± 10.79	99.80 ± 7.90	< 0.001
Waist to hip ratio	0.90 ± 0.07	0.95 ± 0.07	0.96 ± 0.07	0.98 ± 0.07	0.96 ± 0.06	< 0.001
Waist to height ratio	0.58 ± 0.07	0.62 ± 0.07	0.65 ± 0.06	0.65 ± 0.06	0.62 ± 0.05	< 0.001
^$^SBP (mmHg)	113.87 ± 15.74	146.29 ± 16.74	161.94 ± 21.82	168.19 ± 24.07	175.0 ± 19.10	< 0.001
^$$^DBP (mmHg)	73.74 ± 9.40	82.31 ± 11.61	87.56 ± 14.03	96.07 ± 14.07	95.0 ± 18.88	< 0.001
^£^FBS (mg/dl)	99.42 ± 31.83	126.01 ± 49.36	141.30 ± 62.58	143.67 ± 52.54	143.33 ± 59.51	< 0.001**
Median (interquartle rang)	91(18)	107(42)	126(70)	130(78)	128(66)	
Cholesterol (mg/dl)	195.41 ± 39.14	224.39 ± 50.22	217.90 ± 54.44	234.37 ± 67.42	262.420 ± 44.68	< 0.001
Triglyceride (mg/dl)	149.92 ± 81.53	194.61 ± 167.26	181.94 ± 98.48	216.07 ± 138.25	203.42 ± 96.02	< 0.001^**^
Median(interquartle rang)	125(75)	154(109)	158(79)	167(128)	172.5(185)	
^€^LDL (mg/dl)	119.07 ± 33.64	139.21 ± 43.25	131.54 ± 44.87	142.85 ± 61.65	170.92 ± 32.12	< 0.001
^€€^HDL (mg/dl)	46.41 ± 10.96	47.26 ± 12.07	49.96 ± 14.08	48.30 ± 14.80	50.92 ± 19.30	0.02

^€^BMI: Body mass index; ^¥^MET: Metabolic equivalent of task; ^$^SBP: Systolic blood pressure; ^$$^DBP: Diastolic blood pressure;^£^FBS: Fasting blood sugar; ^€^LDL: low density lipoprotein;^€€^HDL: high density lipoprotein;*P: Chi square test; **P: Kruskal Wallis; ^***^P: One Way ANOA

 To explore any correlation of CVD risk with our variables, we ran a logistic regression; the results showed that after adjusting for age and gender, the CVD risk positively correlated with BMI = 25-29.9 kg/m^2^ (OR = 1.70, *P*-value = 0.002) and BMI ≥ 30 kg/m^2^ (OR = 2.60, *P*-value < 0.001). In addition, WHtR ≥ 0.5 (OR = 3.31, *P*-value < 0.001), WHR ≥ 0.9 in males and ≥ 0.85 in females (OR = 2.63, *P*-value < 0.001), and WC ≥ 102 in males and ≥ 88 in females (OR = 1.96, *P*-value < 0.001) were associated with higher CVD risk (Table 3).

**Table 3:** Predictor risk factors of cardiovascular disease according to WHO chart in the Azar cohort population

	**Unadjusted OR (95% CI)**	***P*** **-value **	*Adjusted OR (95%CI)	***P*** ** value **
**Education level**				
Illiterate	7.54(3.96-14.33)	< 0.001	1.72(0.87-3.40)	0.11
Primary school	2.32(1.21-4.45)	0.01	1.61(0.82-3.15)	0.16
Diploma	1.88(0.97-3.67)	0.06	1.24(0.62-2.47)	0.52
University	Reference		Reference	
**Quintiles of wealth index**				
1 (poorest)	2.67(1.87-3.81)	< 0.001	1.13(0.77-1.66)	0.51
2	2.72(1.91-3.89)	< 0.001	1.28(0.87-1.87)	0.19
3	1.49(1.00-2.21)	0.04	0.96(0.64-1.46)	0.88
4	0.67(0.42-1.07)	0.09	0.73(0.45-1.18)	0.20
5 (richest)	Reference			
**Physical activity level (**^$^**METs)**				
Low	1.79(1.39-2.31)	< 0.001	1.06(0.80-1.40)	0.66
Moderate	1.14(0.87-1.50)	0.32	0.95(0.70-1.29)	0.76
High	Reference			
^¥^**BMI (kg/m**^2^**) **				
18.5-24.9	Reference			
< 18.5	1.24(0.29-5.23)	0.76	1.05(0.23-4.67)	0.94
25-29.9	1.40(1.01-1.945	0.04	1.70(1.21-2.39)	0.002
≥ 30	1.94(1.41-2.67)	< 0.001	2.60(1.85-3.65)	< 0.001
^**^WHtR				
< 0.5	Reference			
≥ 0.5	4.16(2.33-7.42)	< 0.001	3.31(1.83-6.00)	< 0.001
^***^WHR				
< 0.9 in males; < 0.85 in females	Reference			
≥ 0.9 in males; ≥ 0.85 in females	4.43(3.14-6.26)	< 0.001	2.63(1.84-3.75)	< 0.001
^€^**WC (cm) **				
< 102 in males; < 88 in females	Reference			
≥ 102 in males; ≥ 88 in females	2.07(1.66-2.587	< 0.001	1.96(1.51-2.53)	< 0.001

^¥^BMI: Body mass index; MET: ^$^Metabolic equivalent of task; **WHtR: Waist to height ratio; ***WHR: Waist to hip ratio; ^€^WC: Waist circumference ^*^Adjusted for age and gender

## Discussion

 The current study aimed to estimate 10-year CVD risk based on the WHO/ISH risk prediction chart among the Azar cohort study population. Our findings demonstrate that 96.8% of the population have a 10-year CVD risk of < 10% and only 0.1% have a CVD risk greater than 40%. Mirzaei et al^14^ also implemented the WHO-EMR B and reported that 83.8% of the population have a ten-year CVD risk of < 10% and 4.2 % greater than 40%. Although the results of the mean age of participants, hypertension prevalence, and cholesterol levels are similar to our study, the variations in the estimated CVD risks are acceptable when considering the actual CVD events. Shabestar is a city in the East Azerbaijan province, Iran, with an adjusted rate of 39.9 myocardial infarctions in a 100,000-population sample.^17^ In contrast, a rate of 141.3 was obtained by Mirzaei et al^14^ in their study conducted in the Yazd.This difference also can explain the dissimilarities in the prevalence of diabetes between the two populations (12.4 % compared to 20.3 % in the Mirzaei et al study).

 In line with the results of the present study, Bavarsad et al yielded 94.1% for the low-risk group and 0.3% for the high-risk group using the WHO-ISH tools for CVD estimation in Shahrekord, Iran.^18^ The prevalence of some risk factors, such as smoking and being overweight, were alike in theirs as well as our study, justifying the consistent results. Another study carried out by Anh Hien et al^19^ that used the WHO/ISH chart in risk prediction showed 5.1% in the high-risk group (> 20%) and 89.8% in the low-risk group (< 10%) in Vietnam. The percentage of people in the low-risk group was less than that obtained in our study, probably due to the younger population in our study. The prevalence of smoking was higher in the Anh Hien et al^19^ study (33.3% in Vietnam and 13.5% in Iran), while the percentage of overweight, obese, and diabetic participants was higher in our study. It seems that differences in lifestyle and ethnicity in each region lead to variations in the contribution of these risk factors in the development of CVD.

 A study undertaken in three low-income Asian countries by Otgontuya et al^20^ used the WHO/ISH risk prediction charts and obtained the following percentages for the low-risk group (< 10%): Cambodia 97%, Malaysia 94.4%, and Mongolia 89%. For the high-risk group (≥ 20%), 1.3%, 2.3%, and 6% were obtained for Cambodia, Malaysia, and Mongolia, respectively. These results are similar to our findings.

 Some studies used specific risk scores for risk assessment based upon extensive cohort studies like the FRS-CVD(Framingham risk score-CVD)^21^ and known risk factors for calculating the 10-year CVD risk. According toprevious studies, these risk factors cannot be employed in populations other than the original for which it was designed, as they can underestimate or overestimate the CVD risk.^14,22-24^

 The results of the present study showed that the prevalence of categorical risk was similar in men and women. However, there are differences in some risk factors between men and women. For example, women have higher BMI, SBP, and cholesterol than men, while men have a higher smoking prevalence. Also, the prevalence of obesity, diabetes, and hypertension washigher in women rather than in men. In accordance with our study, Mirzaei et al^14^ indicated similar results, except the mean of SBP was higher in men.These may result from sex differences in environmental exposures and lifestyle, inequalities in healthcare, and biological differences.^25^ Treatment and control of CVD risk factors were higher in men than in women; that may be because men have, in general, higher CVD risk and, hence, are more likely to meet the initiating treatment than women, and healthcare delivery is less commonly pursued in women than in men, even in the presence of CVD.^26^

 Our findings demonstrated that participants who were overweight and obese had a higher CVD risk. Also, abdominal obesity based on the WHtR, WHR, and WC indices was associated with higher CVD risk. Obesity, especially abdominal obesity as a chronic disorder, is associated with a higher risk of developing non-communicable diseases such as diabetes mellitus, metabolic syndrome, respiratory diseases, hypertension, and heart disease.^27-30^ Therefore, lifestyle and nutrition interventions are required to prevent obesity and CVD.

 As in many developing countries like Iran, regional-specific risk scores are unavailable; hence, WHO recommends using the risk assessment charts provided especially for each region.^8,9^ Unfortunately, these risk scores can easily underestimate the risk byoversimplifying risk factors such as race and BMI, as indicated in our study and many others. The number of people with moderate to high CVD risk was low compared to other regional studies using other risk scores.^18^ While this could be explained in part by different racial, dietary, or geographical factors, the WHO risk score may underestimate the actual risk as its estimates are lower in every study than estimates by FRS-CVDor ASCVD assessment tools.^5,31^ The Asian and Middle Eastern population is afflicted with a high prevalence of central obesity, high dietary salt intake, and etc, not included in the WHO risk charts or other risk scores.^32^ Even though the use of WHO risk charts in comparison to other models (MCCS model, Asian model, Framingham model, or SCORE model) leads to underestimation of the CVD risk,^14,20^ it does not include any blood cholesterol or blood high-density lipoprotein (HDL) cholesterol tests, thus reducing medical costs and making its utilization in practice easier. Although these risk scores are currently recommended, developing region-specific and, more importantly, conclusive risk assessment tools should be prioritized for achieving non-communicable disease goals.

 The large sample size is the main strength of the present study.The major shortcoming is that we applied the risk models of other nations to a portion of the Iranian population. Due to the cross-sectional nature of this study, we were unable to establish a cause-and-effect correlation, and further prospective studies are necessary to establish and affirm such causality.

## Conclusion

 Our results showed that only a small percentage of participants were at high risk of CVD in this Azar cohort study. Moreover, overweight and obese individualshavea higher CVD risk. It warrants lifestyle and nutrition interventions to prevent obesity and CVD. Based on our results, it appears the percentage of people in the high-risk group is underestimated in the WHO/ISH risk prediction charts, leading to delays in receiving appropriate management in the population concerned. Therefore, using other charts alongside the WHO/ISH risk prediction charts is advisable. In addition, we recommended designing a localized WHO prediction chart for use in Iran.

## Acknowledgments

 The authors are grateful for the financial support of the liver and gastrointestinal diseases research center at Tabriz University of Medical Sciences. Theauthors also are deeply indebted to all subjects who participated in this study.We thank the Shabestar Health Center for its close collaboration.In addition, we would like to thank the Persian cohort study staff for theirtechnical support.We appreciate the cooperation of the Clinical Research Development Unit of Imam Reza General Hospital, Tabriz, Iran, in conducting this research.

## Competing Interests

 The authors declare that they have no competing interests.

## Ethical Approval

 The Present study has been approved by the Ethical Committee of the University of Medical Sciences (ethical code: IR.tbzmed.rec.1399.877).
